# 524. Impact of in-vitro Cultured Keratinocyte Allografts on Re-epithelialization Time in Burn Patients

**DOI:** 10.1093/jbcr/irag033.221

**Published:** 2026-04-06

**Authors:** Emanuel Aranda Chavez, Oscar Samaniego Bernal, Ketzarly G Gallardo Duran

**Affiliations:** Instituto Mexicano del Seguro Social, Torreon, Coahuila de Zaragoza; Instituto Mexicano del Seguro Social, Torreon, Coahuila de Zaragoza; IMSS, Torreón, Coahuila de Zaragoza

## Abstract

**Introduction:**

Patients with deep second-degree burns usually require advanced wound management, with meshed split-thickness skin autografts being the technique that provides the best outcomes. However, re-epithelialization often takes up to 18 days or more. In Mexico, in-vitro cultured keratinocyte allografts were developed, and although publications on their use are scarce, it has been reported that they can reduce re-epithelialization time by more than 50%, to an average of 7 days, thereby shortening length of hospital stays and preventing infections, among other potential complications common in this type of patient.

**Methods:**

We aimed to compare the re-epithelialization time of burned wound areas in patients treated with meshed split-thickness skin autografts with and without in-vitro cultured keratinocyte allografts. An observational, analytical, comparative, retrospective cohort study was conducted using medical records of patients admitted to the burn unit from January 1, 2023 to July 31, 2024, with second-degree burns involving less than 40% of the total body surface area. All patients were treated with meshed split-thickness skin autografts and grouped according to whether they additionally received in-vitro cultured keratinocyte allografts.

**Results:**

A total of 39 patients were included; 51.3% (n = 20) were treated with meshed split-thickness skin autografts, and 48.7% (n = 19) received meshed split-thickness skin autografts plus in-vitro cultured keratinocyte allografts. The burn mechanisms were thermal flame (46.2%, n = 18), scald (41%, n = 16), and chemical, radiation, or contact (12.8%, n = 5). The mean burned total body surface area was 9.6 ± 7.5%. The burn wound infection rate was lower in the group treated with meshed split-thickness skin autografts plus in-vitro cultured keratinocytes (26.3% vs 45%, *p*=.224). Re-epithelialization occurred significantly faster in patients who received in-vitro cultured keratinocyte allografts (5.6 ± 2.2 vs 8.3 ± 3.6 days, *p*=.008). Length of hospital stay was also shorter in this group (17.2 ± 8.3 vs 23.9 ± 8.4 days, *p*=.017).

**Conclusions:**

The addition of in-vitro cultured keratinocyte allografts in patients treated with meshed split-thickness skin autografts reduced both re-epithelialization time and length of hospital stay.

**Applicability of Research to Practice:**

The results of this study allow us to determine that the addition of in-vitro cultured keratinocyte allografts enables faster re-epithelialization compared to using only meshed split-thickness skin autografts. This can help patients achieve earlier recovery and reintegration into society, as well as reduce institutional costs associated with prolonged stays in the burn unit and general hospitalization.

**Funding for the study:**

Self funded by the investigators.

